# Genome-Wide Association Studies of Soybean Yield-Related Hyperspectral Reflectance Bands Using Machine Learning-Mediated Data Integration Methods

**DOI:** 10.3389/fpls.2021.777028

**Published:** 2021-11-22

**Authors:** Mohsen Yoosefzadeh-Najafabadi, Sepideh Torabi, Dan Tulpan, Istvan Rajcan, Milad Eskandari

**Affiliations:** ^1^Department of Plant Agriculture, University of Guelph, Guelph, ON, Canada; ^2^Department of Animal Biosciences, University of Guelph, Guelph, ON, Canada

**Keywords:** proximal sensing, support vector machine, hierarchical data integration, soybean breeding, recursive feature elimination (RFE), genome-wide association study (GWAS), multi-omics

## Abstract

In conjunction with big data analysis methods, plant omics technologies have provided scientists with cost-effective and promising tools for discovering genetic architectures of complex agronomic traits using large breeding populations. In recent years, there has been significant progress in plant phenomics and genomics approaches for generating reliable large datasets. However, selecting an appropriate data integration and analysis method to improve the efficiency of phenome-phenome and phenome-genome association studies is still a bottleneck. This study proposes a hyperspectral wide association study (HypWAS) approach as a phenome-phenome association analysis through a hierarchical data integration strategy to estimate the prediction power of hyperspectral reflectance bands in predicting soybean seed yield. Using HypWAS, five important hyperspectral reflectance bands in visible, red-edge, and near-infrared regions were identified significantly associated with seed yield. The phenome-genome association analysis of each tested hyperspectral reflectance band was performed using two conventional genome-wide association studies (GWAS) methods and a machine learning mediated GWAS based on the support vector regression (SVR) method. Using SVR-mediated GWAS, more relevant QTL with the physiological background of the tested hyperspectral reflectance bands were detected, supported by the functional annotation of candidate gene analyses. The results of this study have indicated the advantages of using hierarchical data integration strategy and advanced mathematical methods coupled with phenome-phenome and phenome-genome association analyses for a better understanding of the biology and genetic backgrounds of hyperspectral reflectance bands affecting soybean yield formation. The identified yield-related hyperspectral reflectance bands using HypWAS can be used as indirect selection criteria for selecting superior genotypes with improved yield genetic gains in large breeding populations.

## Introduction

Soybean (*Glycine max* [L.] Merr.) can be considered one of the super crops that is substantially used for food and feed, green manure, biodiesel, and fiber (Seck et al., 2020). Soybean breeders continually breed soybean genotypes with improved desired traits of interest, such as yield (Yoosefzadeh-Najafabadi et al., 2021a). However, yield is a complex trait affected by intrinsic and extrinsic factors as well as their interactions (Anuarbek et al., 2020; Yoosefzadeh-Najafabadi et al., 2021a). Therefore, a sophisticated understanding of the biological aspects of plant genomes is required for sustainable improvements of yield potential in major crops (Somegowda et al., 2021), such as soybean. Soybean breeding programs are moving to implement new genomics, phenomics, and big data analysis for a deeper understanding of soybean yield formation. Having a phenotypic profile of a large plant population with high-density genetic markers are two of the most important factors for better understanding the phenotype and genotype of complex quantitative traits that are usually controlled by various genes with minor and major effects (Wang et al., 2020).

Proximal/remote sensing can be considered as one of the promising high throughput phenotyping tools that can measure the spectral properties of genotypes in a short time in a large breeding population. Most of the spectral measurements are focused on the visible (400–700 nm), red edge (680–780 nm), and near-infrared (700–1100 nm) spectral regions (Alonzo et al., 2014; Hennessy et al., 2020). The visible range can be dissected into blue/blue-green edge (400–499 nm), the green peak (550 nm), and the red (650–700 nm) regions (Rivard et al., 2008; Hennessy et al., 2020). Most of the reflection in the visible region is regulated by the absorption of different foliar pigments such as chlorophyll a and b, carotenoids, and anthocyanins (Castro-Esau et al., 2006; Pu, 2009; Peerbhay et al., 2013; Alonzo et al., 2014; Hennessy et al., 2020). The red-edge region resides between the red and the near-infrared (NIR) regions, which is correlated with internal leaf structure and chlorophyll absorptions (Clevers et al., 2002; Clark et al., 2005; Liu C. et al., 2021). The NIR plateau (780–1327 nm) is another important hyperspectral reflectance region that is dominated by the amount and interaction of water and air within the intercellular spaces (Hennessy et al., 2020; Paulus and Mahlein, 2020; Okubo, 2021).

Several studies reported the high potential of using spectral reflectance to estimate and classify the yield (Yoosefzadeh-Najafabadi et al., 2021a), leaf area index (Chen et al., 2020), plant stress (Feng et al., 2020), and carbon and nitrogen contents (Omidi et al., 2020). In a study done by Zhang et al. (2019), significant association of red and NIR regions with yield are reported. They also demonstrated R5 as the best growth stage for predicting yield and the efficiency of using regression models in predicting soybean yield from the selected hyperspectral reflectance bands. This potential would allow breeders to accurately predict complex traits such as yield, which are typically controlled by several secondary correlated traits, in a short time at early growth stages (Yoosefzadeh-Najafabadi et al., 2021a). While hyperspectral sensors can measure hundreds of wavebands, most of them are redundant due to their high correlation with the adjacent ones (Omidi et al., 2020). Therefore, there is a dire need to find the redundancy of wavebands not only based on the correlation with adjacent bands but also with the estimation of the interaction with other bands in different regions. Genome-wide association studies (GWAS) can be considered as one of the common genetic approaches used for discovering quantitative trait loci (QTL) that are highly associated with a trait of interest (Eltaher et al., 2021). By using GWAS, a QTL associated with a trait of interest can be detected using linkage disequilibrium (LD), which is the non-random association of alleles at specific loci (Somegowda et al., 2021). The detected QTL can be implemented in marker-assisted selection (MAS) for screening large breeding populations in a time- and cost-effective manner (Dababat et al., 2021; Eltaher et al., 2021). Over the past two decades, several statistical methods were used in GWAS to improve statistical power and computational speed (Brachi et al., 2011; Xu et al., 2018). The mixed linear model (MLM) and the fixed and random model circulating probability unification (FarmCPU) approach are known as two of the most common GWAS methods that are currently used in a wide range of genetic studies (Brachi et al., 2011; Lee et al., 2020; Singh et al., 2020). Also, Bonferroni correction and false discovery rate (FDR) are commonly used to set up a threshold for selecting associated QTL with major effects (Brachi et al., 2011; Xu et al., 2018; Lee et al., 2020; Singh et al., 2020).

The application of GWAS was reported in different plant species such as soybean (Brown et al., 2021), maize (Xu et al., 2018), wheat (Tsai et al., 2020), rice (Zhong et al., 2021), and sorghum (Somegowda et al., 2021). While there is no report on the genetic dissection of soybean yield-related hyperspectral reflectance bands, genetic dissection of vegetation index was previously reported in wheat (Wang, 2019; Galán et al., 2020; Wang et al., 2021). Several detected candidate genes related to NDVI, SPAD, and LR in durum wheat (Wang et al., 2021) overlapped with dry biomass, grain yield, and chlorophyll contents. Although GWAS can be considered as a powerful tool to detect the associated genomic regions with major effects, there are several barriers in applying conventional statistical methods in GWAS for identifying genomic regions associated with complex traits (Szymczak et al., 2009). One of the major challenges in GWAS is the high possibility of a false-positive rate that is due to the stochastic noise arise when the population structure is not well defined (Platt et al., 2010) or a high false-negative rate because of the unappropriated way of selecting the threshold (Kaler and Purcell, 2019). Another challenge associated with using the conventional statistical procedures is the “large markers (p), small samples (n)” problem that habitually happens in GWAS when these methods are applied to datasets where the number of markers (i.e., single nucleotide polymorphisms (SNPs)) is significantly larger (*p*≫*n*) than the number of genotypes (Kaler et al., 2020; Mohammadi et al., 2020; Xavier and Rainey, 2020). It is well documented that current conventional GWAS methods are only powerful to detect common SNPs with large effects on the target traits that can reach the minimum level of significance (Lee et al., 2020). Therefore, current conventional GWAS approaches may not be well-suited for discovering minor effect SNPs associated with the target traits, especially in plants with a significantly narrow genetic background (Zhou et al., 2019). For example, several crops, such as soybean, suffer from narrow genetic diversity mainly due to the genetic bottlenecks associated with their domestications and the lack of introducing new sources of genetic diversity (Mikel et al., 2010). While recent advances in sequencing technologies facilitate the accessibility of high-density genetic markers in a short time at a reduced cost, using sophisticated big data analysis methods combined with accurate and rapid large scale phenotyping methods, especially for complex traits, represent major bottlenecks (Hennessy et al., 2020; Yoosefzadeh-Najafabadi et al., 2021a). Recently, Machine Learning (ML) algorithms were shown to be promising computational strategies when applied to plant sciences because of their potential to analyze complex multivariable and nonlinear biological processes, which are commonly observed in complex traits in plants (Jafari and Shahsavar, 2020; Hesami et al., 2021; Yoosefzadeh-Najafabadi et al., 2021a). In general, ML algorithms can be programmed based on existing patterns present in the dataset. Recent studies showed the effectiveness of using ML algorithms to predict complex traits using secondary traits that are highly correlated with the trait of interest (Pantazi et al., 2016; Liakos et al., 2018; Palanivel and Surianarayanan, 2019; Yoosefzadeh-Najafabadi et al., 2021a). Based on the type of problems they solve, ML algorithms can be characterized in four categories as follows: (i) identification, (ii) classification, (iii) quantification, and (iv) prediction. These four categories could be used to identify important variables from multi-dimensional datasets (Liakos et al., 2018; Hesami et al., 2020; Sharifi, 2021). Variable selection methods are commonly used to improve the prediction performance and avoid overfitting rates for classification and prediction problems in high-dimensional datasets (George, 2000). The variable selection methods are, in general, classified into three distinct groups: wrappers, filters, and embedded methods (George, 2000; Heinze and Dunkler, 2017). In filter methods, subsets of variables are selected based on selection criteria that are independent from those used for the final classifier (Chowdhury and Turin, 2020). However, both wrapper and embedded methods implement variable selection based on individual learners (Albashish et al., 2021). For example, recursive feature elimination (RFE) is a representative wrapper-type variable selection algorithm widely used to extract important features from phenomics and genomics data (Gupta and Gupta, 2020; Albashish et al., 2021; Yoosefzadeh-Najafabadi et al., 2021a). RFE discards in an iterative fashion the weak and unstable variables until a target number of variables is reached and thus retains independent variables from the dataset resulting in significant improvements in performance and reduced overfitting of ML algorithms (Sanz et al., 2018).

In addition to the proper ML algorithm choice, adopting an accurate data integration strategy is required for a better understanding of the structure of complex multidimensional traits at different omics levels (Tarazona et al., 2021). These days, more and more data are generated using different omics such as genomics and phenomics, and several data integration strategies such as early, intermediate, late, mixed, and hierarchical strategies are available (Jamil et al., 2020; Picard et al., 2021). A hierarchical data integration strategy is built upon prior knowledge about the relationship between and among different tested omics layers (Picard et al., 2021). For instance, a hierarchical data integration strategy can be used for a better understanding of soybean yield formation by having prior knowledge about the physiological concept of each hyperspectral reflectance in explaining the overall yield variation. The effectiveness of using RFE was reported previously by Yoosefzadeh-Najafabadi et al. (2021a) to extract the important wavelengths for predicting soybean seed yield. In addition, a few studies used ML algorithms in GWAS for detecting QTL associated with complex traits (Zhou et al., 2019; Xavier and Rainey, 2020; Najafabadi et al., 2021). In a GWAS study, Xavier and Rainey (2020) investigated the potential use of Random Forest (RF) for detecting QTL associated with soybean yield components, such as the number of pods and nodes. In addition, the use of RF for detecting QTL with minor effects was reported in a study by Asif et al. (2020). However, using other promising ML algorithms such as support vector regression (SVR) and implementing a hierarchical data integration strategy for better understanding soybean yield using hyperspectral and genome-wide association studies is long overdue. Therefore, this study aimed to: (1) investigate the use of RFE for selecting hyperspectral reflectance wavelengths influencing soybean yield, (2) evaluate the potential use of SVR- mediated GWAS for genetic dissection of important hyperspectral reflectance bands affecting soybean yield, and (3) discover candidate genes linked to identified hyperspectral reflectance bands associated with yield. To the best of our knowledge, this study is the first report where GWAS was used to discover hyperspectral reflectance bands associated with soybean yield. This study also demonstrates the benefits of using ML algorithms and variable selection methods in phenome-phenome and phenome-genome association studies for discovering yield-related physiological traits and genomic regions associated with these traits in soybean. The results of this study can be useful for selecting high yielding soybean genotypes at early growth stages and, therefore, increasing the rate of genetic gain for yield in cultivar development programs.

## Materials and Methods

### Genome-Wide Association Studies Panel and Experimental Design

The GWAS panel was consisted of 227 diverse soybean genotypes that were grown and evaluated for the target traits in Ridgetown (42°27′14.8″N 81°52′48.0″W, 200 m above sea level) and Palmyra (42°25′50.1″N 81°45′06.9″W, 195 m above sea level), Ontario, Canada, over two consecutive years, 2018 and 2019. The experimental design was conducted based on the randomized complete block design (RCBD) in four environments (two locations × two years) with two replications in each environment. Each phenotypic plot for each genotype was consisted of five rows, each 4.2 m long with a row spacing and seedling rate of 43 cm and 57 per m^2^, respectively. Overall, there were 1000 soybean plots per year and 500 soybean plots per environment. Also, nearest-neighbor analysis (NNA), as one of the most common error control methods, was used to estimate the accuracy of the phenotypic evaluations and control the spatial variability in the field (Stroup and Mulitze, 1991; Bowley, 1999; Katsileros et al., 2015).

### Seed Yield and Hyperspectral Reflectance Data Collection

Soybean seed yield (t ha^–1^) was estimated for each plot after harvesting of three middle rows and adjusting for day to maturity and the seed moisture to 13%.

Previous studies reported that environmental stresses at the seed development growth stage (R5), where seeds are 1/8 inches long in pods at one of the four uppermost nodes (Yoosefzadeh-Najafabadi et al., 2021a), in compared to other growth stages, could have greater damage to the soybean yield. It can be because of the fact that plants have no time to recover the yield before physiological maturity (Zhang et al., 2019; Yoosefzadeh-Najafabadi et al., 2021a). Therefore, the R5 growth stage in soybean can be considered as a reliable growth stage for measuring hyperspectral reflectance, if the goal is to predict the overall seed yield (Zhang et al., 2019; Yoosefzadeh-Najafabadi et al., 2021a). The hyperspectral reflectance was measured at the beginning of the R5 stage. Hyperspectral reflectance bands were measured via UniSpec-DC Spectral Analysis System (PP Systems International, Inc., 110 Haverhill Road, Suite 301 Amesbury, MA, United States), which covers 250 bands from 350 to 1100 nm with a 3 nm bandwidth. In general, the measured hyperspectral reflectance consisted of three main regions: visible, red-edge, and near-infrared (NIR) regions. Spectralon panels and a dark reference background were used to adjust incoming solar radiation and calibrate the dual channels, respectively. For each plot, three measurements were recorded at the same spot in order to reduce the noise, and their average, calculated by the best linear unbiased prediction (BLUP) model, was used as the reflectance band datapoint. All of the measurements were conducted close to solar noon to reduce the signal-to-noise (SNR) ratio.

### Hyperspectral Data Pre-processing

The pre-processing step is one of the most important steps in hyperspectral reflectance analysis, which reduces the possible electronic fluctuations and sensor noises in datasets. By checking the quality of hyperspectral reflectance data for the tested panel and the result of sensor-specific artifacts, hyperspectral reflectance data of 1,005–1,100 and 350–395 nm, were removed from the original data. The number of reflectance bands was decreased from 250 bands to 62 by increasing the bandwidth from 3 to 10 nm. In order to improve the signal-to-noise ratio, a Savitzky–Golay filter was applied for each reflectance band and data scaling, centering, and principal component analysis (PCA) was conducted in order to detect potential outliers in the dataset. All the pre-processing steps were performed using R software (version 3.6.1).

### Statistical Analyses

In order to estimate the genetic values of each soybean genotype, the BLUP was used as one of the most well-known mixed models (Goldberger, 1962). For this aim, ‘environment’ and ‘genotype’ factors were considered as fixed and random effects, respectively. Based on the protocol developed by Bowley (1999), all outliers were detected and treated the same as missing data points in further analysis. Overall, the statistical model used in this study is as follows:


(1) $$Y=X_{b}+Z_{g}+W_{i}+e$$

where Y is the vector of trait of interest (selected hyperspectral reflectance bands), b is the vector of block effects, encompasses all the replications and locations, added to the overall mean (assumed fixed), g in the vector of genotype effects (assumed random), in which g ∼ N(0, σ^2^_g_), i is the vector of random GxE interaction effects, in which i ∼ N(0, σ^2^_i__nt_), and *e* is the vector of residuals, in which e ∼ N(0, σ^2^_*e*_). X, Z, and W represent the incidence matrices of b, g, and i effects, respectively.

Also, the heritability (Eq. 2) of each tested trait was calculated based on the following equation:


(2) $$H^{2}=\frac{\sigma_{g}^{2}}{\sigma_{g}^{2}+\sigma_{int/n}^{2}+\sigma_{e/nr}^{2}}$$

where σ^2^*g* is the genotypic variance; σ^2^*int* is the variance of GxE; σ^2^*e* is error variance; n is the number of locations; r is the number of replications.

### Hyperspectral Wide Association Study

With respect to the genome-wide association study, we proposed the term of hyperspectral wide association study (HypWAS) for detecting hyperspectral reflectance bands associated with the trait of interest. In order to detect the important hyperspectral reflectance bands associated with the trait of interest, RFE, as one of the most common variable selection methods (Guyon et al., 2002), was used in this study. The main basis of the RFE is to eliminate variables with low importance scores and select the high importance score variables that explain the trait of interest. In RFE, the first step is to build a model on the complete set of the inputs and computing the importance of each input based on sequential selection strategy (Guyon et al., 2002). The next step is to remove the least important inputs and rebuilding the model to recursively repeat the process. In general, RFE shows how important is a feature for a model with respect to predicting a value and it is strictly describing the prediction power of a feature. In this study, we implemented RFE, considering reflectance bands as input variables and soybean yield as an output variable. All the analyses were done using the *caret* package (Kuhn, 2008) in R software version 3.6.1.

### Genotyping

For extracting DNA, young trifoliate leaf tissue was collected from the first soybean phenotyping plot of each genotype at the Ridgetown location and stored after freeze-drying using the Savant ModulyoD Thermoquest (Savant Instruments, Holbrook, NY, United States). DNA was isolated using NucleoSpin Plant II kit (Macherey–Nagel, Duüren, Germany) as per the manufacturer’s instructions, and the quality of DNA was checked with Qubit^®^ 2.0 fluorometer (Invitrogen, Carlsbad, CA, United States). The extracted DNA were sent to Genomic Analysis Platform at Université Laval (Laval, Quebec, Canada) for genotyping-by sequencing (GBS) based on the enzymatic digestion with *ApeKI* (Sonah et al., 2013). GBS for each genotype was done via the Fast-GBS pipeline (Torkamaneh et al., 2020), using Gmax_275_v2 reference genome. After imputing the missing loci by the Markov model using Beagle v5 pipeline and removing markers with a minor allele frequency less than 0.05, a total of 17,958 high-quality single-nucleotide polymorphisms (SNPs) from 227 soybean genotypes used for genomic analysis.

### Population Structure Analysis

A total of 17,958 high-quality SNPs were used to conduct the population structure analysis using fastSTRUCTURE (Raj et al., 2014) with K values from 1 to 15. Afterward, the optimum number of subpopulations was calculated using the K tool in the fastSTRUCTURE software.

### Association Studies

In this study, MLM and FarmCPU, as the two conventional GWAS methods, were compared with the developed SVR-mediated GWAS method. All the conventional GWAS methods were implemented using the *MVP* (Yin et al., 2021) package in R software version 3.6.1. The popular *Caret* package (Kuhn et al., 2020) in R, was used to develop the SVR-mediated GWAS method.

#### Mixed Linear Model

One of the most common methods for GWAS is the MLM method developed by Yu et al. (2006). This method has been widely used in GWAS because of its effectiveness in controlling the bias in the population and correcting the inflation from different small genetic effects caused by polygenic background (Bulik-Sullivan et al., 2015; Wang S.-B. et al., 2016; Wen et al., 2018). While the likelihood ratio is not specific to the MLM method, this method is based on the likelihood ratio between the full model (with a marker of interest) and the reduced model (without a marker of interest) (Wen et al., 2018). If we considered Y as the phenotypic value, the MLM equation would be as follows (Eq. 3):


(3) $$Y=X_{b}+Z_{u}+e$$

where Y is the vector of phenotypic observations; b is the vector of SNP markers and population structure effects (assumed fixed); u is the vector of additive genetic effects for genotypes (assumed random); e is the vector of residuals (assumed random). X and Z represent the incidence matrices of b and u effects, respectively.

#### Fixed and Random Model Circulating Probability Unification

This GWAS method was first introduced by Liu et al. (2016) in order to reduce the shortcoming and false discoveries that existed in previously proposed GWAS methods. FarmCPU takes advantage of using the random-effect (REM) and fixed-effect (FEM) models iteratively (Liu et al., 2016). In brief, FEM was used to test the S number of SNPs, simultaneously, based on the following equation (Eq. 4):


(4) $$Y_{i}=S_{i1}B_{1}+S_{i2}B_{2}+S_{i3}B_{3}+\ldots +S_{it}B_{t}+M_{ij}K_{j}+e_{i}$$

Where Y_*i*_ stands for the observation on the ith sample, S_*i1*_, S_*i2*_,…, S_*i*__*t*_ stand for the genotypes of the t pseudo-QTNs, B_1_, B_2_, B_3_, …, B_*t*_ is the corresponding effect for the pseudo-QTNs, M_*ij*_ is the genotype of the jth SNPs and ith sample, K_*j*_ is known as the corresponding effect of the jth SNPs, and e_*i*_ is the residual.

The REM model is used in the FarmCPU method to optimize the selection of the genetic markers based on the *p*-values as follows (Eq. 5):


(5) $$Y_{i}=U_{i}+e_{i}$$

Where Y_*i*_ stands for the observation on the ith sample, e_*i*_ is the residual, and U_*i*_ is the total genetic effect of the ith sample.

Also, false discovery rate (FDR) was used for MLM and FarmCPU to set the significant threshold (Benjamini and Hochberg, 1995).

#### Support Vector Regression

Support vector regression represents a support vector machine (SVMs) approach used to solve regression problems (Awad and Khanna, 2015). SVR is characterized by the use of the Vapnik-Chervonenkis (VC) theory, sparse solution, and kernels for controlling the number of vectors and margin (Smola and Schölkopf, 2004; Awad and Khanna, 2015). This algorithm is trained by implementing an asymmetrical loss function that equally penalizes low and high misestimates (Vapnik, 1998). The association statistics for SVR can be obtained by evaluating the feature importance, which is previously proposed by Weston et al. (2001). In this study, SNPs were considered as inputs, and the selected hyperspectral reflectance bands were selected as output variables for evaluating the feature importance using the SVR algorithm. In brief, the following equation was used to determine SVR (Eq. 6):


(6) Y=Wβ(c)+b

Where Y is the output, W stands for the weights for each high dimensional input (β) that is constructed non-linearly on the input space of (c). The upper and lower borderlines are presented as *Y* = *W*β(*c*) + *b* + *e*+ and *Y* = *W*β(*c*) + *b*-*e*, respectively.

The five-fold cross-validation strategy (Siegmann and Jarmer, 2015) was used to run the variable importance analysis with ten repetitions. The impurity index was selected as the common metric to evaluate the importance of each SNP in explaining the trait of interest. After implementing the variable importance, the achieved scores were scaled to a 0–100% scale. After fitting the algorithm, high variable importance was stored during 1000 times repetitions. Then, all significant SNPs were selected based on a confidence level α = 0.05. The global empirical threshold was used for estimating the significant threshold of SNPs associated with selected hyperspectral reflectance bands (Churchill and Doerge, 1994; Doerge and Churchill, 1996).

### Extracting Candidate Genes Underlying Detected Quantitative Trait Locis

**FIGURE 1:**
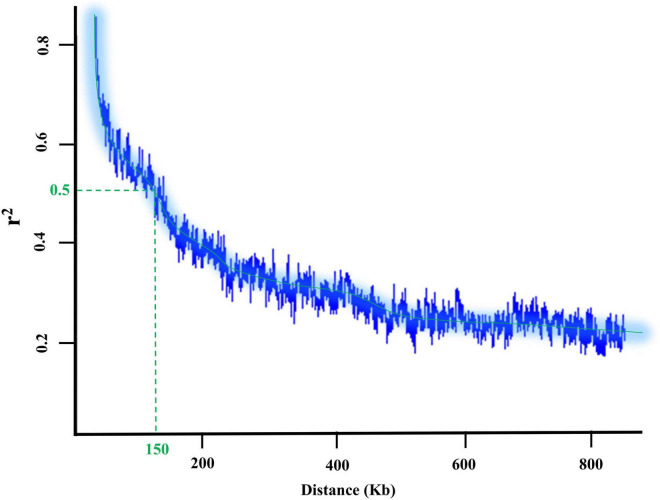
LD decay plot and the flanking regions of each detected SNP in 227 soybean genotypes.

The potential candidate genes for the tested GWAS methods were extracted from the *Glycine max* William 82 reference gene models 2.0 using the SoyBase database^1^. The flanking regions of associated peak SNPs with the trait of interest were obtained based on the LD decay distance (Figure 1). Also, Gene Ontology (GO) enrichment analysis (see text footnote 1), and previous studies were used to detect genes associated with the trait of interest and investigate their system biology functions for each trait. Finally, the Electronic Fluorescent Pictograph (eFP) browser for soybean^2^ and transcriptomics data from Severin et al. (2010) were included to generate further information about the candidate genes, including developmental- and tissue-stage dependent gene expression levels.

### Data Integration Strategy

**FIGURE 2:**
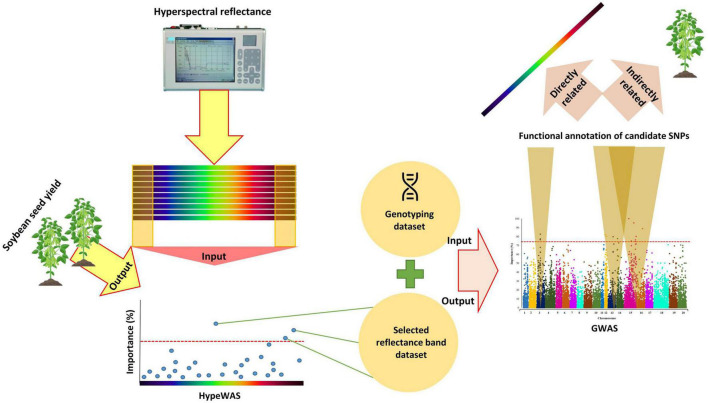
The schematic workflow of the hierarchical data integration strategy of HypWAS and GWAS in Soybean.

In this study, the hierarchical data integration strategy was used to accommodate the using the of HypWAS results as prior knowledge for GWAS analyses. By using a hierarchical data integration strategy, genomic regions that are directly associated with the selected hyperspectral reflectance bands and indirectly related to the overall soybean yield can be detected. Afterward, the associated candidate genes with the tested hyperspectral reflectance bands can be identified based on the results of the GWAS analysis (Figure 2).

## Results

### Yield Statistics and Hyperspectral Reflectance Profile

**FIGURE 3:**
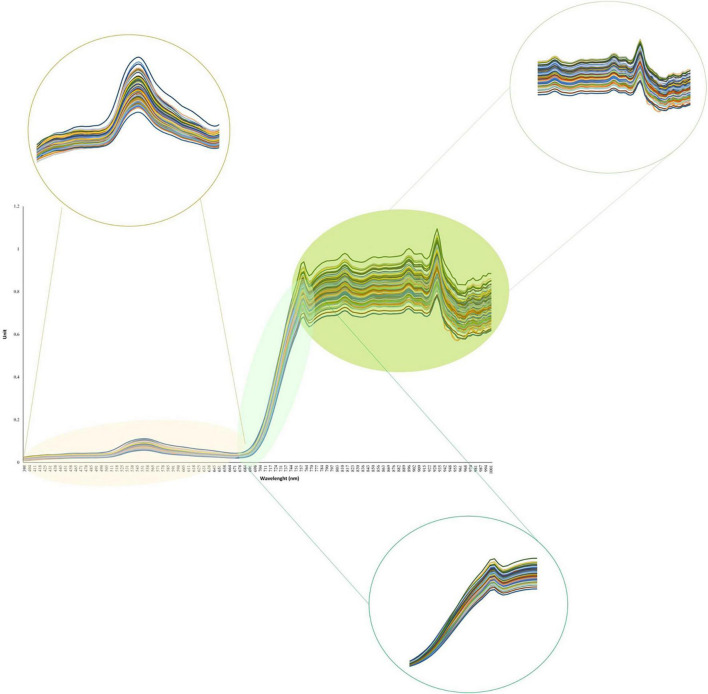
The hyperspectral reflectance profile of the tested 227 soybean genotypes.

The average seed yield for all soybean genotypes evaluated in four environments ranged from 2.6 to 5.7 t ha^–1^ with a standard deviation and mean of 0.57 and 4.22 t ha^–1^, respectively. The results of analysis of variance for yield as well as heritability are represented in Supplementary Table 1. Overall, the heritability of yield in the tested panel was 0.24. The complete hyperspectral reflectance profile measured for the tested soybean panel is presented in Figure 3. The visible and NIR regions showed the highest variation among the genotypes with a range of 0.40 and 0.56, respectively, whereas the red-edge region had the lowest variation among all the hyperspectral regions with a range of 0.11 (Figure 3). Within the visible region, the highest variations were present in the green, and red regions ranged from 0.12 and 0.13, respectively, while other reflectance bands in the visible region had lower variations (Figure 3). The reflectance bands greater than 770 nm showed larger variations among soybean genotypes compared to reflectance bands in the red-edge region (Figure 3). The results of analysis of variances for each selected hyperspectral reflectance band are presented in Supplementary Tables 2–6. Among all the tested hyperspectral reflectance bands, 660 and 730 nm had the highest and lowest heritability with values of 0.85 and 0.28, respectively (Supplementary Tables 2–6). All the selected reflectance bands showed a significant difference among genotypes.

### Hyperspectral Wide Association Study

**FIGURE 4:**
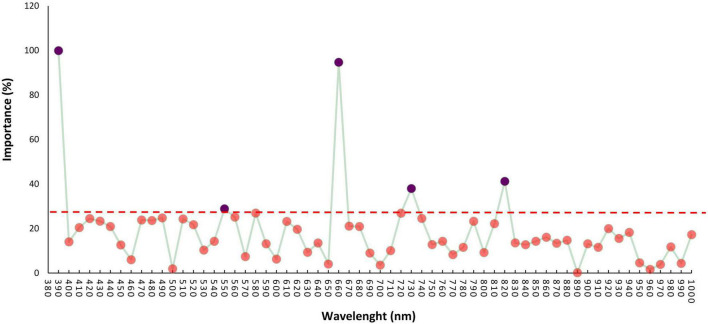
Hyperspectral wide association analysis (HypWAS) of yield in the tested soybean panel. The threshold for significant importance values was indicated as the red dash line.

**FIGURE 5:**
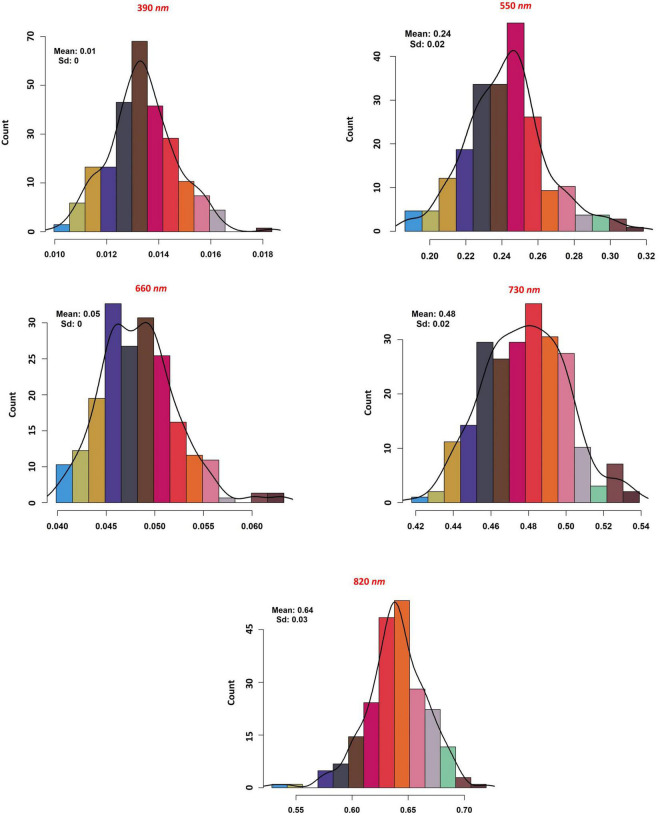
The distribution, mean and standard deviation of the selected hyperspectral reflectance bands in the tested soybean panel.

The association between reflectance bands and soybean seed yield was assessed using the RFE method. Among all the 62 reflectance bands, five were found to be significantly associated with the soybean yield (Figure 4). The selected reflectance bands were 390, 550, 660, 730, and 820 nm with the importance score of 99, 29, 94, 38, and 41%, respectively. Based on the number of important reflectance bands, the visible region was the most informative reflectance region associated with soybean yield by having three out of five important bands, namely, 390, 550, and 660 nm (Figure 4). Among all the reflectance bands located in the red-edge and NIR regions, the 730 and 820 nm bands were associated with soybean seed yield in the red-edge and NIR regions, respectively (Figure 4). Only the selected reflectance bands were chosen for further analysis. As it can be seen in Figure 5, all selected reflectance bands had a normal distribution. The 390 nm, as the hyperspectral reflectance band with the highest importance score, had a mean of 0.1 in the tested panel across different environments. The second hyperspectral reflectance band with high importance score was the 660 nm band with a mean of 0.05 (Figure 5). As the lowest importance score among all the selected hyperspectral reflectance bands, the 550 nm band had the mean and standard deviation of 0.24 and 0.02, respectively (Figure 5).

**FIGURE 6:**
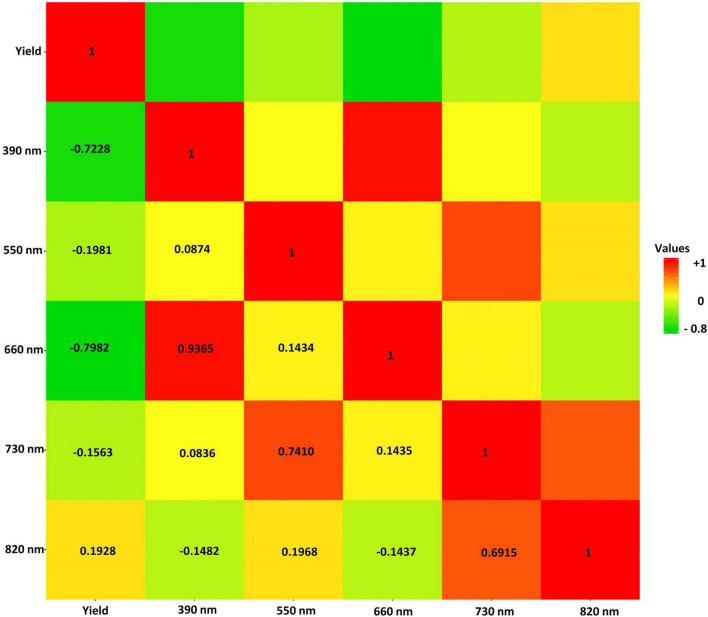
The Pearson correlation of the selected hyperspectral reflectance bands in the tested soybean panel. The heat map scale for values is provided by color for the panel.

Pearson Product-Moment Correlation coefficients of all the selected reflectance bands with soybean yield were estimated and are shown in Figure 6. The only positive correlation with yield was found in 820 nm band with the *r* = 0.19 (Figure 6). The highest negative correlation with yield was observed in the 660 nm band with a correlation coefficient (*r*) of −0.80, and the lowest negative correlation was found between the 730 nm band and seed yield with *r* = −0.16 (Figure 6).

### Genotyping Evaluations

**FIGURE 7:**
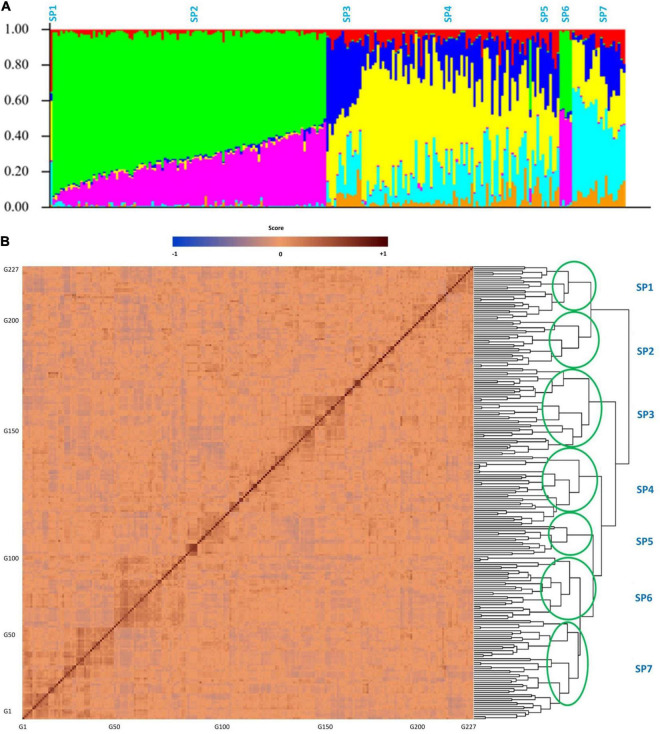
Structure **(A)** and kinship **(B)** plots for the 227 soybean genotypes. The *x*-axis represents the number of genotypes used in this GWAS panel, and the *y* axis represents the membership of each subgroup. SP1–SP7 stands for subpopulations 1 to 7.

High-quality SNPs were obtained for the tested GWAS panel from 210M single-end Ion Torrent reads that were proceeded with Fast-GBS.v2. After the filtering process, 17,958 out of 40,712 SNPs were detected as polymorphic and then mapped onto 20 soybean chromosomes. In the tested GWAS panel, the maximum number of SNPs was 1780 on chromosome 18, and the minimum number of SNPs was 403 on chromosome 11. The average number of SNPs was 898 across all the 20 chromosomes, with the mean density of one SNP for every 0.12 cM across the genome. As illustrated in Figure 7A, the tested soybean panel was composed of four to seven subpopulations. Therefore, the *K* = 7 was used as the optimum K for the tested association panel. Also, the kinship was calculated between soybean genotypes to reduce the confounding effect (Figure 7B).

### Genome-Wide Association Studies Analysis

**FIGURE 8:**
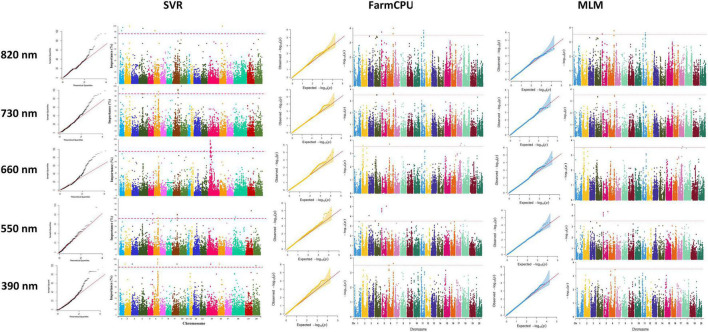
Genome-wide Manhattan plots for GWAS studies of the selected hyperspectral reflectance bands using MLM, FarmCPU, and SVR methods in the tested soybean panel. MLM, mixed linear model; FarmCPU, fixed and random model circulating probability unification; SVR, fixed and random model circulating probability unification.

In this study, we conducted GWAS analysis for dissecting the genetic control of the yield-related reflectance and identified SNPs that are linked to selected bands from HypWAS. According to the GWAS analysis of the 390 nm band, using the MLM method, 10 SNPs located on chromosomes 3, 6, and 10 were found to be associated with this band (Figure 8). Using FarmCPU, 13 SNPs on chromosomes 3, 6, 10, and 13 were identified, and exploiting the SVR-mediated GWAS, 12 SNPs on chromosomes 2, 3, 6, 9, 15, 16, and 20 were found to be linked to this band (Figure 8). Based on the results, chromosomes 3 and 6 are two chromosomes that were found associated with the 390 nm band using all three GWAS methods. Most of the detected QTL on chromosomes 3 and 6 were co-localized with previously reported QTL such as ureide content, resistance to *Phytophthora sojae*, internode length, and pubescence color (Table 1).

**TABLE 1:** The list of detected QTLs for 390 nm using different GWAS methods in the tested soybean panel.

GWAS Method	Chromosome	Peak SNP position	Co-located QTL	Environment^a^	References
MLM	3	44121448	Ureide content 1-g13.1	NA	Ray et al., 2015
			Ureide content 1-g13.2	NA	Ray et al., 2015
			Ureide content 1-g13.3	NA	Ray et al., 2015
			Ureide content 1-g13.4	NA	Ray et al., 2015
			Ureide content 1-g13.5	NA	Ray et al., 2015
		44198005		NA	
		44326068		NA	
		44328629		NA	
		45039908		NA	
	
	6	11953156		NA	
		11953223		NA	
	
	10	2906452		NA	
		2906459		NA	
		2906496		NA	

FarmCPU	3	44121448	Ureide content 1-g13.1	NA	Ray et al., 2015
			Ureide content 1-g13.2	NA	Ray et al., 2015
			Ureide content 1-g13.3	NA	Ray et al., 2015
		44198005	Ureide content 1-g13.4	NA	Ray et al., 2015
			Ureide content 1-g13.5	NA	Ray et al., 2015
		44326068		NA	
		44328629		NA	
		45039908	Internode length 1-g3	NA	Fang et al., 2017
			Pubescence color 2-g1.1	NA	Fang et al., 2017
	
	6	11953223		NA	
		11953223		NA	
	
	10	2906452		NA	
		2906459		NA	
		2906496		NA	
		50548092		NA	
	
	13	30231980	Seed Yield 3-g5	NA	Contreras-Soto et al., 2017
		30232000	seed weight 6-g2	2	Sonah et al., 2015
			Phytoph 3-g22	2	Chang et al., 2016
			Phytoph 2-g4	2	Qin et al., 2017
			Phytoph 2-g5	2	Qin et al., 2017
			Phytoph 3-g23	2	Chang et al., 2016
			Leaflet width 1-g1	2	Fang et al., 2017

SVR	2	858431		NA	
		858458		NA	
	
	3	3729245	Phytoph 3-g1	NA	Chang et al., 2016
			Phytoph 3-g2	NA	Chang et al., 2016
			Phytoph 3-g3	NA	Chang et al., 2016
			Phytoph 3-g4	NA	Chang et al., 2016
			Phytoph 3-g5	NA	Chang et al., 2016
			Phytoph 3-g6	NA	Chang et al., 2016
	
	6	38679976		NA	
		38787915		NA	
		36746367		NA	
	
	9	39344365	pod number 1-g4.1	NA	Fang et al., 2017
			pod number 1-g4.2	NA	Fang et al., 2017
		39344439	pod number 1-g4.3	NA	Fang et al., 2017
			seed thickness 2-g4	NA	Fang et al., 2017
	
	15	22232022		NA	
		22231908		NA	
	
	16	7313753	Sclero 3-g60	NA	Moellers et al., 2017
			ureide content 1-g43	NA	Ray et al., 2015
	
	20	22656999		NA	

*^*a*^Detected in separate environments in addition to the combined environment. (1) 2018Ridgetown, (2) 2019Ridgetwon, (3) 2018Palmyra, (4) 2019Palmyra, (NA) Not found in any separate environment.*

*MLM, mixed linear model; FarmCPU, fixed and random model circulating probability unification; RF, random forest; SVR, support vector regression.*

Genome-wide association studies analyses on the 550 nm band resulted in discovering 5 and 10 SNPs to be associated with this band using the MLM and the FarmCPU methods, respectively. Among all the associated SNPs using the MLM method, four of them were located on chromosome 5, and one on chromosome 3. The 10 SNPs identified using the FarmCPU method were located on chromosomes 1, 3, 5, and 13 (Figure 8). Using SVR-mediated GWAS analysis, we identified nine SNPs linked to the 550 nm band, of which two were located on chromosome 6, three on chromosome 9, and one SNP on each of the chromosomes 5, 15, 18, and 19, respectively (Figure 8). Comparing the results of the three GWAS methods, chromosome 5 was consistently found to be associated with the 550 nm band. Most of the detected QTL on chromosome 5 were co-localized with previously reported QTL such as oil-related traits, water use efficiency, and full maturity (Table 2).

**TABLE 2:** The list of detected QTLs for 550 nm using different GWAS methods in the tested soybean panel.

GWAS Method	Chromosome	Peak SNP position	Co-located QTL	Environment^a^	References

MLM	3	22283256		NA	
	
	5	1115619	seed palmitic 4-g1	2	Zhang et al., 2018
			seed palmitic 4-g1.2	2	Zhang et al., 2018
			Seed long chain fatty acid 1-g21.2	2	Fang et al., 2017
			Seed long chain fatty acid 1-g19.2	2	Fang et al., 2017
		1115673	Seed long chain fatty acid 1-g14.1	2	Fang et al., 2017
			Seed long chain fatty acid 1-g14.2	2	Fang et al., 2017
		1115689	Seed long chain fatty acid 1-g1.2	2	Fang et al., 2017
			seed stearic 3-g1	2	Zhang et al., 2018
		41767154	Phytoph 2-g28	2	Qin et al., 2017
			First flower 4-g18	2	Mao et al., 2017
			Seed oil 4-g18	2	Bandillo et al., 2015
			Seed oil 4-g17	2	Bandillo et al., 2015
			Seed oil 4-g16	2	Bandillo et al., 2015
			Seed oil 6-g1	2	Cao et al., 2017
			Seed oil 6-g16	2	Cao et al., 2017
			R8 full maturity 5-g1	2	Fang et al., 2017
			Seed oil 4-g15	2	Bandillo et al., 2015
			Seed oil 4-g14	2	Bandillo et al., 2015
			Seed oil 6-g2	2	Cao et al., 2017
			Seed oil 6-g4	2	Cao et al., 2017

FarmCPU	1	41281098			
	
	3	22283184			
		22283256			
		44326068		4	
	
	5	1115619	Seed palmitic 4-g1	2	Zhang et al., 2018
			Seed palmitic 2-g1.2	2	Fang et al., 2017
			seed long chain fatty acid 1-g21.2	2	Fang et al., 2017
		1115673	seed long chain fatty acid 1-g19.2	2	Fang et al., 2017
		1115673	seed long chain fatty acid 1-g14.1	2	Fang et al., 2017
		1115689	seed long chain fatty acid 1-g14.2	2	Fang et al., 2017
		1153958	seed long chain fatty acid 1-g1.2	2	Fang et al., 2017
		1115689	seed stearic 3-g1	2	Zhang et al., 2018
		41767154	First flower 4-g18	2	Mao et al., 2017
			seed oil4-g18	2	Bandillo et al., 2015
			seed oil 4-g17	2	Bandillo et al., 2015
			seed oil 4-g16	2	Bandillo et al., 2015
			seed oil 6-g1	2	Cao et al., 2017
			seed linolenic 4-g5	2	Li et al., 2015
			R8 full maturity 5-g1	2	Fang et al., 2017
			seed oil 4-g15	2	Bandillo et al., 2015
			seed oil 4-g14	2	Bandillo et al., 2015
			seed oil 6-g2	2	Cao et al., 2017
			seed oil 8-g4	2	Zhang et al., 2018
	
	13	29190399	Phytoph 2-g30	2	Qin et al., 2017
			seed set 1-g29-.2	2	Fang et al., 2017
			seed weight 13-g8	2	Wang J. et al., 2016

SVR	5	40467080	WUE 2-g13	3	Kaler et al., 2017
			Shoot p 1-g10.2	3	Dhanapal et al., 2018
			shoot p 1-g10.1	3	Dhanapal et al., 2018
	
	6	41477920		4	
		38960003		NA	
	
	9	39366957	Pod number 1-g4.1	1	Fang et al., 2017
			Pod number 1-g4.2	1	Fang et al., 2017
			Pod number 1-g4.3	1	Fang et al., 2017
			Seed thickness 2-g4	1	Fang et al., 2017
		39372117	Seed Thr 2-g1	1	Li et al., 2018
			Seed Sar 2-g1	1	Li et al., 2018
			Seed Tyr 2-g1	1	Li et al., 2018
		39659468	Seed Lys 2-g1	NA	Li et al., 2018
			Seed Leu 2-g1	NA	Li et al., 2018
			Seed LIu 2-g1	NA	Li et al., 2018
			Seed Ala 2-g1	NA	Li et al., 2018
			Seed Gly 2-g1	NA	Li et al., 2018
	
	15	11293240	Seed protein 7-g14	NA	Zhang et al., 2017
	
	18	11166966		NA	
	
	19	36064225		NA	

*^*a*^Detected in separate environments in addition to the combined environment. (1) 2018Ridgetown, (2) 2019Ridgetwon, (3) 2018Palmyra, (4) 2019Palmyra, (NA) Not found in any separate environment.*

*MLM, mixed linear model; FarmCPU, fixed and random model circulating probability unification; RF, random forest; SVR, support vector regression.*

According to Figure 8, a total of 9, 14, and 15 associated SNPs were detected using MLM, FarmCPU, and SVR, respectively. Out of 9 detected SNPs using the MLM method, four SNPs were located on chromosome 3, three SNPs were located on chromosome 7, and two SNPs were located on chromosome 18 (Figure 8). All the detected SNPs using FarmCPU were located on chromosomes 3, 6, and 18 (Figure 8). GWAS analysis of the 660 nm band using the SVR-mediated GWAS method detected 13 SNPs on chromosome 15 and one SNP on chromosomes 14 and 19 (Figure 8). Most of the detect QTL for the 660 nm band were related to first flower, water use efficiency, soybean cyst nematode resistance, seed yield, pod number, and plant height (Table 3).

**TABLE 3:** The list of detected QTLs for 660 nm using different GWAS methods in the tested soybean panel.

GWAS Method	Chromosome	Peak SNP position	Co-located QTL	Environment^a^	References

MLM	3	22283184		NA	
		22283218		NA	
		22283256		NA	
		44326068		NA	
	
	7	20220381		NA	
		20280812		NA	
		23349322		NA	
	
	18	56952847	pod number 4-g8	NA	Hao et al., 2012
			plant height 3-g14	NA	Contreras-Soto et al., 2017
		56952858	shoot K 1-g39	NA	Dhanapal et al., 2018
			WUE 3-g32	NA	Dhanapal et al., 2018

FarmCPU	3	22283184		NA	
		22283218		NA	
		22283256		NA	
		44326068		NA	
	
	7	20220330	Shoot Mn 1-g2	NA	Dhanapal et al., 2018
		20220381		NA	
		20280812		NA	
		23317163	Ureide content 1-g9	NA	Ray et al., 2015
		23349322	shoot Mn 1-g3	NA	Dhanapal et al., 2018
		27492738		NA	
	
	18	56952847	pod number 4-g8	NA	Hao et al., 2012
			plant height 3-g14	NA	Contreras-Soto et al., 2017
		56952858	shoot K 1-g39	NA	Dhanapal et al., 2018
			WUE 3-g32	NA	Dhanapal et al., 2018
		57794992		NA	

SVR	14	16425108		NA	
	
	15	13118545		NA	
		12895268		NA	
		12894320		NA	
		14174744		NA	
		14378690		1	
		12892003	seed coat color 3-g3	NA	Vuong et al., 2015
			seed yield, soyNAM 7-g14	NA	Diers et al., 2018
		18302021		NA	
		14406716		1	
		17877705		NA	
		17362699		NA	
		14088239		1	
		13163194	SCN 5-g33	NA	Li et al., 2016
			First flower 4-g58	NA	Mao et al., 2017
			First Flower 5-g29.1	NA	Fang et al., 2017
			First flower 5-g29.2	NA	Fang et al., 2017
			First flower 5-g29.3	NA	Fang et al., 2017
		14421366		1	
	
	19	36064225		1	

*^*a*^Detected in separate environments in addition to the combined environment. (1) 2018Ridgetown, (2) 2019Ridgetwon, (3) 2018Palmyra, (4) 2019Palmyra, (NA) not found in any separate environment.*

*MLM, mixed linear model; FarmCPU, fixed and random model circulating probability unification; RF, random forest; SVR, support vector regression.*

Genome-wide association studies analyses on the 730 nm band resulted in discovering 10, 15, and 10 associated SNPs using MLM, FarmCPU, and SVR, respectively (Figure 8). Using MLM, 10 SNPs were located on chromosomes 1, 2, 5, 14, 15, 17, and 18 (Figure 8). By using the FarmCPU method, five associated SNPs with the 730 nm band were located on chromosome 5, three SNPs were located on chromosome 2, two SNPs were on chromosomes 1 and 18, and one SNP was located on chromosomes 14, 15, and 17 (Figure 8). Using SVR-mediated GWAS, 10 SNPs were located on chromosomes 1, 4, 6, 9, and 10 (Figure 8). Based on these results, chromosome 1 was unanimously determined to be associated with the 730 nm band by all three GWAS methods. Most of the detected QTL for the 730 nm band were related to water use efficiency, seed oil and protein-related traits, reproductive stage length, and pod number (Table 4).

**TABLE 4:** The list of detected QTLs for 730 nm using different GWAS methods in the tested soybean panel.

GWAS Method	Chromosome	Peak SNP position	Co-located QTL	Environment^a^	References

MLM	1	41281098		NA	
	
	2	17694706		NA	
		17694726		NA	
	
	5	1115689	Seed palmitic 4-g1	NA	Zhang et al., 2018
			Seed palmitic 2-g1.2	NA	Fang et al., 2017
			Seed long-chain fatty acid 1-g21.2	NA	Fang et al., 2017
		1153958	Seed long-chain fatty acid 1-g19.2	NA	Fang et al., 2017
			Seed long-chain fatty acid 1-g14.1	NA	Fang et al., 2017
			Seed long-chain fatty acid 1-g14.2	NA	Fang et al., 2017
			Seed long-chain fatty acid 1-g1.2	NA	Fang et al., 2017
			Seed stearic 3-g1	NA	Zhang et al., 2018
	
	14	2259506	Reproductive stage length 1-g3.1	NA	Fang et al., 2017
			Reproductive stage length 1-g3.2	NA	Fang et al., 2017
	
	15	9020829		NA	
	
	17	31797213		NA	
	
	18	6686269		NA	
		6728432	Seed protein 7-g27	NA	Zhang et al., 2017

FarmCPU	1	16343505		NA	
		41281098		NA	
	
	2	17694706		NA	
		17694726		NA	
		42645195	WUE 2-g6	4	Kaler et al., 2017
			WUE 3-g4	4	Dhanapal et al., 2018
			WUE 3-g5	4	Dhanapal et al., 2018
			WUE 3-g6	4	Dhanapal et al., 2018
	
	5	1115619	Seed palmitic 4-g1	NA	Zhang et al., 2018
			Seed palmitic 2-g1.2	NA	Fang et al., 2017
			Seed long-chain fatty acid 1-g21.2	NA	Fang et al., 2017
		1115673	Seed long-chain fatty acid 1-g19.2	NA	Fang et al., 2017
			Seed long-chain fatty acid 1-g14.1	NA	Fang et al., 2017
		1115689	Seed long-chain fatty acid 1-g14.2	NA	Fang et al., 2017
			Seed long-chain fatty acid 1-g1.2	NA	Fang et al., 2017
		1153958	Seed stearic 3-g1	NA	Zhang et al., 2018
		1467115	Seed palmitic 5-g2	NA	Li et al., 2015
			Seed palmitic 5-g1	NA	Li et al., 2015
			Seed palmitic 2-g1.3	NA	Fang et al., 2017
			Seed long-chain fatty acid 1-g21.3	NA	Fang et al., 2017
			Seed long-chain fatty acid 1-g19.3	NA	Fang et al., 2017
			Seed long-chain fatty acid 1-g1.3	NA	Fang et al., 2017
			Seed width to height ratio 1-g2.1	NA	Fang et al., 2017
			Seed width to height ratio 1-g2.2	NA	Fang et al., 2017
			Seed width to height ratio 1-g2.3	NA	Fang et al., 2017
	
	14	2259506	Reproductive stage length 1-g3.1	NA	Fang et al., 2017
		2259506	Reproductive stage length 1-g3.2	NA	Fang et al., 2017
	
	15	9020829	Seed coat luster 1-g1.1	NA	Fang et al., 2017
			WUE 3-g26	NA	Dhanapal et al., 2018
			WUE 3-g27.1	NA	Dhanapal et al., 2018
	
	17	31797213		NA	
	
	18	6686269	Seed protein 7-g27	NA	Zhang et al., 2017
		6728432	Seed protein 7-g27	NA	Zhang et al., 2017

SVR	1	47953926		1	
	
	4	19007585		NA	
		18643026		NA	
	
	6	41477920		NA	
	
	9	39659468	Seed Ser 2-g1	2	Li et al., 2018
			Seed Thr 2-g1	2	Li et al., 2018
		39664525	Seed Tyr 2-g2	NA	Li et al., 2018
			Seed Lys 2-g2	NA	Li et al., 2018
			Seed Leu 2-g2	NA	Li et al., 2018
			Seed Ala 2-g2	NA	Li et al., 2018
			Seed Gly 2-g2	NA	Li et al., 2018
		39366957	Pod number 1-g4.1	NA	Fang et al., 2017
			Pod number 1-g4.2	NA	Fang et al., 2017
		39372117	Pod number 1-g4.3	NA	Fang et al., 2017
			seed thickness 2-g4	2	Fang et al., 2017
		40355403		NA	
	
	10	3054709		NA	

*^*a*^Detected in separate environments in addition to the combined environment. (1) 2018Ridgetown, (2) 2019Ridgetwon, (3) 2018Palmyra, (4) 2019Palmyra, (NA) Not found in any separate environment.*

*MLM, mixed linear model; FarmCPU, fixed and random model circulating probability unification; RF, random forest; SVR, support vector regression.*

Using MLM, FarmCPU, and SVR methods for GWAS analysis of the 820 nm band, a total of four, five, and seven SNPs were detected to be associated with this reflectance band. The associated SNPs were located on each of the chromosomes 1, 4, 6, and 16 using MLM (Figure 8). Using the FarmCPU method, two associated SNPs were found on chromosome 16, and one was found on chromosomes 1, 5, and 6, respectively (Figure 8). Two associated SNPs using the SVR-mediated GWAS method were located on chromosome 1, and one SNP was found on chromosomes 2, 4, 6, 10, and 16 (Figure 8). Chromosomes 1, 6, and 16 were selected as the commonly detected chromosomes among all the tested GWAS methods associated with the 820 nm band. Most of the detected QTL for the 820 nm band were related to first flower, soybean cyst nematode resistance, water use efficiency, seed set, seed long-chain fatty acid, and seed width to height (Table 5).

**TABLE 5:** The list of detected QTLs for 820 nm using different GWAS methods in the tested soybean panel.

GWAS Method	Chromosome	Peak SNP position	Co-located QTL	Environment^a^	References

MLM	1	16343505		NA	
	
	5	1467115	seed palmitic 5-g2	3	Li et al., 2015
			seed palmitic 5-g1	3	Li et al., 2015
			seed palmitic 2-g1.3	3	Fang et al., 2017
			seed long-chain fatty acid 1-g19.3	3	Fang et al., 2017
			seed long-chain fatty acid 1-g1.3	3	Fang et al., 2017
			seed long-chain fatty acid 1-g21.3	3	Fang et al., 2017
			seed width to height ratio 1-g2.1	3	Fang et al., 2017
			seed width to height ratio 1-g2.2	3	Fang et al., 2017
			seed width to height ratio 1-g2.3	3	Fang et al., 2017
	
	6	50187091	Sclero 3-g32	3	Moellers et al., 2017
	
	16	36071599	Seed set 1-g21.2	NA	Fang et al., 2017
			Seed set 1-g21.1	NA	Fang et al., 2017
			First Flower 4-g66	NA	Mao et al., 2017
			SCN 5-g38	NA	Li et al., 2016

FarmCPU	1	16343505		NA	
	
	5	1467115	seed palmitic 5-g2	3	Li et al., 2015
			seed palmitic 5-g1	3	Li et al., 2015
			seed palmitic 2-g1.3	3	Fang et al., 2017
			seed long-chain fatty acid 1-g19.3	3	Fang et al., 2017
			seed long-chain fatty acid 1-g1.3	3	Fang et al., 2017
			seed long-chain fatty acid 1-g21.3	3	Fang et al., 2017
			seed width to height ratio 1-g2.1	3	Fang et al., 2017
			seed width to height ratio 1-g2.2	3	Fang et al., 2017
			seed width to height ratio 1-g2.3	3	Fang et al., 2017
	
	6	50187091	Sclero 3-g32	3	Moellers et al., 2017
	
	16	36071599	Seed set 1-g21.2	NA	Fang et al., 2017
			Seed set 1-g21.1	NA	Fang et al., 2017
		36236254	First Flower 4-g66	NA	Mao et al., 2017
			SCN 5-g38	NA	Li et al., 2016

SVR	1	39990647		NA	
		47953926		NA	
	
	2	42411031	WUE 3-g-2	4	Dhanapal et al., 2018
			WUE 3-g-3	4	Dhanapal et al., 2018
			WUE 2-g6	4	Kaler et al., 2017
			WUE 3-g4	4	Dhanapal et al., 2018
			WUE 3-g5	4	Dhanapal et al., 2018
	
	4	20000416		NA	
	
	6	11029779		NA	
	
	10	3054709		3,4	
	
	16	17376063		NA	

*^*a*^Detected in separate environments in addition to the combined environment. (1) 2018Ridgetown, (2) 2019Ridgetwon, (3) 2018Palmyra, (4) 2019Palmyra, (NA) not found in any separate environment.*

*MLM, mixed linear model; FarmCPU, fixed and random model circulating probability unification; RF, random forest; SVR, support vector regression.*

### Extracting Candidate Genes Underlying Detected Quantitative Trait Locis

**FIGURE 9:**
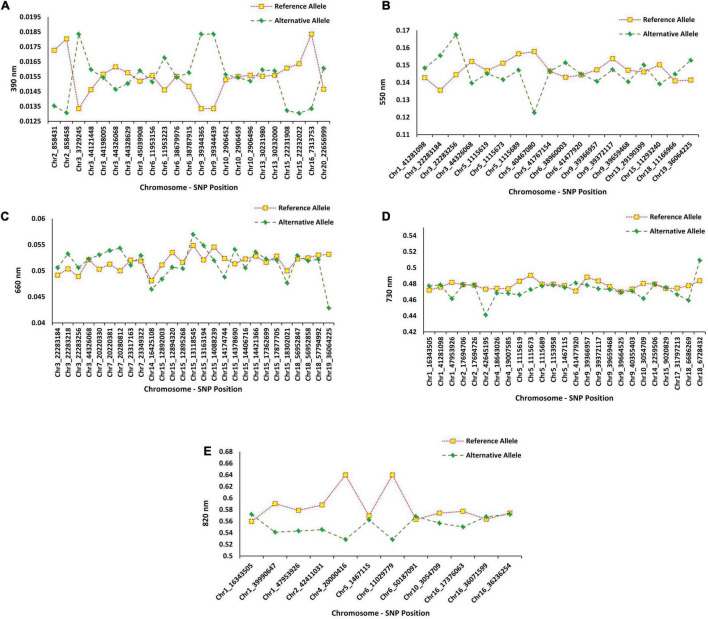
The average effects of reference allele and alternative allele from the detected SNP’s peak for **(A)** 390 nm, **(B)** 550 nm, **(C)** 660 nm, **(D)** 730 nm, and **(E)** 820 nm in 227 soybean genotypes across four environments.

The flanking regions of the QTL were determined within the 150-kbp upstream and downstream of each peak SNP for a given QTL, and these regions were searched for identifying potential candidate genes associated with the target bands (Figure 1). For the 390 nm band, five peak SNPs (Chr2_858458, Chr3_3729245, Chr9_39344365, Chr9_39344439, and Chr16_7313753) had the highest allelic effects (Figure 9A). Based on the gene annotation and expression data, the following genes were identified as selected candidates governing the 390 nm band: *Glyma.02G008900* (GO:0006979), *Glyma.02G008700* (GO:0006970), *Glyma. 03G033100* (GO:0019748), *Glyma.03G033100* (GO:0031347), *Glyma.09G168700* (GO:0009624 and GO:0042742), and *Glyma.16G073100* (GO:0050660 and GO:0016491). These genes have been annotated for oxidative and osmotic stresses, secondary metabolic process, regulation of defense response, response to nematode, defense response to bacterium, flavin adenine dinucleotide binding, and oxidoreductase activity, respectively. For the 550 nm band, two peak SNPs (Chr3_22283256 and Chr5_40467080) had the highest allelic effects compared to other detected peak SNPs (Figure 9B). There was no previously reported QTL linked with Chr3_44326068, while three QTL (water use efficiency and shoot macro- and micronutrient concentrations) were linked to Chr5_40467080. The candidate genes *Glyma.03G081700* (GO:0010224) and *Glyma.05G226000* (GO:0009411, GO:0009813) were selected candidates for the 550 nm band, which encode response to UV-B and to UV, and flavonoid biosynthetic process, respectively. Based on Figure 9C, the highest allelic effect for the 660 nm band was found in one peak SNP (Chr19_36064225) detected by the SVR-mediated GWAS, whereas no previously reported QTL was linked to the detected peak SNP position. Based on the gene ontology analysis, *Glyma.19G108200* (GO:0009911, GO:0048573, and GO:0009909) was the selected candidate gene for the 660 nm band, which encodes positive regulation of flower development, photosynthesis, flowering, light reaction, and regulation of flower development. The allelic effect analysis of the 730 nm band indicated a high allelic effect for two peak SNPs at Chr2_42645195 and Chr18_6728432 (Figure 9D). The selected peak SNPs were linked to water use efficiency and seed protein contents (Figure 9D). There were two selected candidate genes [*Glyma.02G237700* (GO:0009853) and *Glyma.02G237400* (GO:0031347)] associated with the 730 nm band, which encode photorespiration and regulation of defense response. On the 820 nm band, the highest allelic effect was found in two peak SNPs (Chr4_20000416 and Chr6_11029779) that were detected by the SVR-mediated GWAS method (Figure 9E). Gene ontology analysis of the selected peak SNPs identified two candidate genes, Glyma.04G135300 (GO:0009658 and GO:0009055) and Glyma.10G033600 (GO:0015250 and GO:0005215), which encode chloroplast organization, electron carrier activity, water channel activity, and transporter activity, respectively.

## Discussion

Accurate predictions of the final performance of germplasm at early growth stages is paramount for breeders to make early selection decisions in germplasm advancement and cultivar development programs (Maimaitijiang et al., 2020). The end-season selection based on yield *per se* may result in overlooking other factors such as selecting for other yield-unrelated traits and environmental factors (Yoosefzadeh Najafabadi, 2021). Yet, trait measurement in large breeding nurseries and working with large phenotypic and genotypic data is still a bottleneck in the genome-to-phenome analysis process (Parmley et al., 2019). In recent years, the combination of high throughput phenotyping and genotyping tools has greatly accelerated the plant breeding progress (Yang et al., 2020). However, merging different omics datasets for better characterization of the complex traits extensively depends on an appropriate selection of a data integration strategy (Picard et al., 2021). Extracting the biological information from the secondary related traits that are in high correlation with the trait of interest, detecting the associated SNPs, and identifying candidate genes for each detected peak SNPs provided a valuable complement for understanding the biological mechanism of a trait of interest. The hierarchical strategy is based on including prior knowledge of relationships between different omics layers (Wang et al., 2013; Picard et al., 2021). In this study, we used this strategy in a soybean hyperspectral reflectance-yield association study to acquire a better understanding of the genetic architecture of soybean yield using GWAS.

As one of the important high-throughput phenotyping tools, hyperspectral reflectance has provided plant breeders with an efficient plant evaluation strategy in a large population at early growth stages for important agronomic traits (Yoosefzadeh-Najafabadi et al., 2021a). The use of spectral reflectance for predicting crop yield has been extensively investigated. For example, Qiao et al. (2021) reported the efficiency of using long-time series multi-spectral images for yield mapping of different crop species using an automated spatial-spectral feature extractor. The application of hyperspectral reflectance in predicting the wheat grain yield was studied by Fei et al. (2021), who reported the effectiveness of red and NIR regions in predicting the grain yield in different irrigation regimes. The use of hyperspectral reflectance in predicting yield was not limited to agronomy crops and used for vegetables (Awika et al., 2021), trees (Ali and Imran, 2021), and industrial plants (Holmes et al., 2020). Therefore, breeders can use spectral data to establish phenome-to-genome relationships by performing HypWAS and applying it via phenomics selection. Genomic selection methods suffer from the lack of consideration for environmental effects (Zhong et al., 2009; Tong and Nikoloski, 2021). However, by using HypWAS, both environmental and genetic effects can be considered in the final decision. The proposed idea of HypWAS is novel, and, to our best knowledge, there is no example in the literature.

The rationale behind the HypWAS is to increase the efficiency of indirect selection for breeders and to offer them a strategy to reduce noise and possible errors from the hyperspectral reflectance data that is used in genetic studies. HypWAS makes it possible to select reflectance bands with high importance scores for complex traits such as yield. Therefore, plant breeders and geneticists can investigate more aspects of the selected reflectance bands to find the genomic regions, gene candidates, and physiological processes behind each reflectance band. Previously, we implemented one of the most common variable selection methods, RFE, to select important reflectance bands in association with soybean yield production (Yoosefzadeh-Najafabadi et al., 2021a). However, there was no further information about the genetic background of the selected reflectance bands. In this study, we selected the five most important reflectance bands and we used HypWAS to investigate various aspects of the physiological and genetic background of each reflectance band.

Among the five selected reflectance bands, three of them were located in the visible range of the spectrum. Most of the reflection in the visible region is significantly dominated by the foliar pigments’ absorption (Hennessy et al., 2020). Chlorophyll a and b have a stronger reflection in comparison with other foliar pigments in the visible region (Fernandes et al., 2013). Chlorophylls play a major role in photosynthesis due to all the photosynthesis structures, such as the antenna systems of photosystem I and photosystem II (Pettai et al., 2005; Hennessy et al., 2020). Light energy absorbed by antenna systems of photosystem I and II, is then rapidly transferred to the respective reaction centers (Pettai et al., 2005). Several studies reported the strong correlation of the 680–700 nm bands with photosystem I and II (Ke, 2001; Pettai et al., 2005; Hoa et al., 2017; Hennessy et al., 2020). In this study, the 680 nm band was selected as one of the high-importance reflectance bands that explains the total soybean seed yield. This can reveal the importance of photosynthesis components in determining the overall soybean yield (Yoosefzadeh-Najafabadi et al., 2021c). The 660 nm band can be used to screen a large population of plants in a short time for selecting genotypes with a high potential for photosynthesis activity. Similarly, another important reflectance band in this study (390 nm) is located in the blue region of the spectrum, which is highly correlated with the chlorophyll content (Richter et al., 2016; Hennessy et al., 2020). The 390 nm band had the highest importance value in predicting the final soybean yield among all the selected reflectance bands, confirming the importance of photosynthesis in the final soybean yield formation. The third detected reflectance band in this study (550 nm), represents the green peak in the visible spectrum (Stommel et al., 2009; Liu et al., 2018). This reflectance band was reported to be correlated with Chlorophyll and Anthocyanin contents in plants (Hennessy et al., 2020). Anthocyanins are known as a diverse class of flavonoid components that play a significant role in protecting plants against abiotic and biotic stresses (Gitelson et al., 2001; Hennessy et al., 2020). Since most of the soybean fields in North America are grown in rainfed areas, water deficit stress would be inevitable during the soybean growing season in these areas.

Many studies reported the strong correlation between red-edge and near-infrared regions and water content as well as spongy mesophyll conditions in plants. Based on the HypWAS analysis, the 730 and 820 nm bands were selected as the important reflectance bands in predicting the overall soybean yield. The 730 nm band is located in the red-edge region and correlated with the leaf water content, chlorophyll concentration, and leaf layering (Horler et al., 1983). The 820 nm band is located in the near-infrared region and reflects the spongy mesophyll condition in leaves (Gao et al., 2014; Salvatori et al., 2015). The major role of spongy mesophyll in plants is to interchange the required CO_2_ for photosynthesis (Veromann-Jürgenson et al., 2020). All spongy mesophylls are covered by a thin layer of water, hence environmental stresses can significantly affect mesophylls resulting in a reduced photosynthesis activity level in plants (Veromann-Jürgenson et al., 2020; Liu M. et al., 2021). Therefore, changes in the water and gas level in mesophylls is the first sign of detecting stresses in plants, so the difference between the reflectance of the 730 nm as well as the 820 nm band in normal and stress conditions can be considered as a measurement for abiotic and biotic stresses (Momayyezi et al., 2020). Overall, adjusting breeding selection criteria based on the selected reflectance bands might lead to select a genotype with significant levels of tolerance against stresses and high photosynthesis activity. This can be done by measuring those reflectance bands by remote sensing tools in a short time in a less labor-intensive manner. Also, understanding the genetic background of each selected reflectance band would be helpful to design appropriate genetic markers for the fast screening of genotypes.

Genome-wide association studies is currently considered as an imperative approach for discovering genomic regions associated with complex traits in diverse areas from human genetics to plant and animal breeding (Alqudah et al., 2020; Khanzadeh et al., 2020; Li et al., 2020; Tibbs Cortes et al., 2021). Insufficient statistical power is the most fundamental challenge when it comes to using conventional GWAS for characterizing quantitative traits (Nicholls et al., 2020), especially in plants with narrow genetic bases. In ML-mediated GWAS analyses, the significance levels or thresholds for identifying SNP-trait associations are estimated using variable importance methods, which are different from statistical methods that are used for estimating *p*-value in conventional GWAS (Szymczak et al., 2009). The main advantage of using variable importance, rather than *p*-values, for individual SNP-trait association tests, consists in the ability of these approaches to consider the interaction effects between SNPs (Szymczak et al., 2009; Asif et al., 2020). In other words, ML algorithms are more practical in terms of allowing for high-order interactions that are not pre-specified in the model using non-linear kernels (Sun et al., 2021). Conventional statistical methods need to have pre-identified parameters for the analysis of special traits of interest (Sun et al., 2021). Conventional statistical methods are significantly useful in the presence of inherent uncertainty, small signal-to-noise ratio, insufficient training dataset, a small number of variables, predefining the parameters involved in the variance of the trait of interest. Therefore, conventional GWAS are appropriate approaches for detecting SNPs with large main effects on complex traits. However, they are underpowered to simultaneously consider a wide range of interconnected biological processes and mechanisms that shape the phenotype of complex traits (Lee et al., 2020). By using ML algorithms in GWAS, the interaction and joint effect of multiple SNPs can be estimated using variable importance methods, and the best set of SNPs will be selected to give the best performance (Pahikkala et al., 2012). Recent studies showed that the SNPs with high importance scores are not necessarily the SNPs with significant *p*-values resulted from single SNP analyses (Arshadi et al., 2009; Grömping, 2009; Szymczak et al., 2009; Ziliak, 2017; Di Leo and Sardanelli, 2020). Therefore, using variable importance values estimated by ML algorithms for identifying SNP-trait associations may improve the power of ML-mediated GWAS for discovering variant-trait associations with higher resolution (Szymczak et al., 2009). The variable importance methods based on linear and logistic regressions, Support vector machines, and random forest algorithms are well established in the literature (Grömping, 2009; Wu and Liu, 2009; Chun and Keleş, 2010; Williamson et al., 2020; Yoosefzadeh-Najafabadi et al., 2021b). In this study, we found that SVR-mediated GWAS had the same performance in detecting numbers of QTL when compared to conventional GWAS methods. However, the detected QTL by SVR-mediated GWAS was more related to the physiological background of each tested hyperspectral reflectance bands. For instance, in the 820 nm band, the SVR-mediated GWAS detected 5 QTL related to water use efficiency, which is clearly in agreement with the physiological background of this trait. The same scenario happened in the 660 nm band, where most of the detected QTL by SVR-mediated GWAS were related to flowing and soybean cyst resistance. In all the tested hyperspectral reflectance bands, several QTL related to the soybean seed protein, oil, pod number, seed yield, and seed thickness were detected in all GWAS methods. Meanwhile, seed protein, oil, pod number, seed yield, and seed thickness can be considered as the yield component traits, which directly and indirectly regulate the final soybean seed yield. Therefore, the detected QTL confirmed the efficiency of HypWAS and GWAS in indirect selection for complex traits such as yield.

Furthermore, several candidate genes were detected by SVR-mediated GWAS related to the oxidative and osmotic stresses, regulation of defense response, response to nematode, defense response to bacterium, and oxidoreductase activity. It is well documented that the violet spectrum and UV radiation are key factors in secondary metabolite production (e.g., terpenes, alkaloids, phenolic compounds, glucosinolates, and carotenoids) that can play a pivotal role in a plant’s defense systems (Schreiner et al., 2012; Matsuura et al., 2013). It has also been shown that these spectra lead to the activation of several signaling pathways such as defense signaling, reactive oxygen species (ROS), and photomorphogenic signaling (Schreiner et al., 2012; Matsuura et al., 2013). These signaling can stimulate and induce the specific gene expression patterns involved in different secondary metabolism pathways, such as the isoflavonoid biosynthesis pathway (Kim et al., 2014). MYB family is one of the most important transcriptional factors that may interact with light-responsive elements and thereby activate selected genes involved in isoflavonoid biosynthesis (Du et al., 2010). Moreover, a positive correlation was reported between the expression profiles of the selected genes (*Glyma.02G008700* and *Glyma.09G168700*) and the patterns of isoflavonoid accumulation, which shows the biosynthesis of isoflavonoid might be activated by violet spectra through the up-regulation of these genes (Du et al., 2010). The spatiotemporal regulation of chlorophyll metabolism is necessary for various cellular processes such as chloroplast development, photosynthesis, plastid-derived retrograde signaling (Chan et al., 2016), RNA metabolism (Zhang et al., 2014), singlet oxygen-mediated signaling (Shen et al., 2006), abscisic acid signaling, and programmed cell death (Woodson et al., 2015; Dogra et al., 2019). Chlorophyll metabolism can be categorized into four functional classes including (i) Chlorophyll a synthesis through the branched tetrapyrrole biosynthesis pathway (Tanaka and Tanaka, 2007; Mochizuki et al., 2010), (ii) the ‘Chlorophyll cycle,’ which catalyzes the interconversion of Chlorophyll a and Chlorophyll b (Tanaka and Tanaka, 2011), (iii) the degradation of Chlorophyll a to yield colorless through the pheophorbide a oxygenase (PAO)/phyllobilin pathway (Christ and Hörtensteiner, 2014), and (iv) Chlorophyll recycling pathway through dephytylase 1 (CLD1) (Lin et al., 2016). The combination of divinyl reductase (DVR) and light-dependent protochlorophyllide oxidoreductase (POR) produces chlorophyllide (Chlide) a. Subsequently, Chlorophyll synthase (CHLG) catalyzes Chlorophyll a biosynthesis through the combination of Chlide a and phytyl pyrophosphate (phytyl-PP) (Wang and Grimm, 2021). CLD1 can reversibly convert Chlorophyll a into Chlide a during Chlorophyll recycling. Also, it is well documented that high-light inducible proteins (Hlips) play an important role in binding Chlorophyll a and β-carotene and consequently the photosynthesis capacity (Chidgey et al., 2014; Staleva et al., 2015; Shukla et al., 2018).

It has been well documented that three main families of carbonic anhydrase (CA) genes, including α-CAs, β-CAs, and γ-CAs, play essential roles in the conductance of inorganic carbon through carbon fixation rates and the mesophyll (de Araujo et al., 2014; Cano et al., 2019). They may also be involved in sensing light, CO2, and water availability (Momayyezi et al., 2020). Therefore, CAs can affect photosynthetic efficiency through their impacts on stomatal response to light, CO2-facilitating components (aquaporins), ABA signaling, and other signaling pathways (de Araujo et al., 2014; Cano et al., 2019; Momayyezi et al., 2020). ABA is a key phytohormone associated with stomatal closure. ABA receptors (e.g., PYL, PYR, RCAR proteins) play an important role in executing ABA’s function in water relations (Cutler et al., 2010; Kim et al., 2010). ABA regulates the stress-activated kinase signaling network that controls stomatal closure (Mega et al., 2019). In reacting to water deficit, the level of ABA increases, which regulates the ligand-receptor complex formation that represses the clade A protein phosphatase 2Cs (PP2Cs) activity, which is considered negative regulators for ABA signaling (Fujii et al., 2009; Ma et al., 2009; Park et al., 2009). Because of the central role of ABA receptors in transpiration regulation, they can be considered as promising targets for breeding programs in order to manipulate ABA sensitivity and water productivity (Mega et al., 2019).

## Conclusion

Indirect selection of complex traits would be of paramount importance in analytical breeding strategies. Nowadays, the use of advanced high throughput phenotyping and genotyping combined with big data analysis methods can ease the assessment of large plant breeding populations in a very effective short time. For the first time in this study, we are proposing the HypWAS method for identifying hyperspectral reflectance bands associated with complex traits such as yield. Based on this method, we were able to discover, five hyperspectral reflectance bands significantly associated with the soybean seed yield. The visible region of the spectra was found to be the most informative region related to the seed yield. The GWAS analyses of the selected hyperspectral reflectance bands using MLM, FarmCPU, and a newly developed SVR-mediated GWAS method revealed several QTL revealing the bands that seem to be related to the soybean seed yield, water use efficiency, and soybean cyst nematodes resistance based on previous studies. In general, all of the tested GWAS methods had acceptable performance. However, we were able to detect more relevant QTL using the SVR-mediated GWAS. Regarding the Gene Ontology of the selected traits, most of the detected genes were reported to be related to the water status, photosynthesis, and light intensity. The obtained genetic results confirmed the physiological background of the selected hyperspectral reflectance bands. The result of this study can be used to accelerate the indirect breeding selection strategy for selecting high-yielding genotypes based on specific hyperspectral reflectance bands at early plant growth stages. In addition, the genetic results can be employed to use the detected QTL in each hyperspectral reflectance band for MAS selection in large breeding populations.

## Data Availability Statement

The datasets presented in this study can be found in online repositories. The names of the repository/repositories and accession number(s) can be found below: https://github.com/Mo hsen1080/Available-Datasets/blob/92f27c80fa3e460900589b4218 8a943570dee86d/FastGBS.SNPs.232.imputed.Het50.maf0.05.hm p.txt, GitHub.

## Author Contributions

ME conceptualized, designed and directed the experiments. MY-N conducted the experiments, modeled, summarized the results, and writing the manuscript. ST participated in candidate gene analyses. ST, DT, IR, and ME revised the manuscript and validated the results. All authors have read and approved the final manuscript.

## Conflict of Interest

The authors declare that the research was conducted in the absence of any commercial or financial relationships that could be construed as a potential conflict of interest.

## Publisher’s Note

All claims expressed in this article are solely those of the authors and do not necessarily represent those of their affiliated organizations, or those of the publisher, the editors and the reviewers. Any product that may be evaluated in this article, or claim that may be made by its manufacturer, is not guaranteed or endorsed by the publisher.

## Footnotes

1
https://www.soybase.org

2
www.bar.utoronto.ca
