# Supplementary material for: Genome-Wide Association Studies of Soybean Yield-Related Hyperspectral Reflectance Bands Using Machine Learning-Mediated Data Integration Methods
Source: Front Plant Sci. 2021 Nov 22;12:777028. doi: 10.3389/fpls.2021.777028 (PMC8647880; doi:10.3389/fpls.2021.777028)
Supplement: Supplementary file 1 [file Table_1.DOCX]

**Genome-Wide Association Analyses of Soybean Yield-Related Hyperspectral Reflectance Bands Using Machine Learning-Mediated Data Integration Methods**

**Mohsen Yoosefzadeh-Najafabadi,^1^ Sepideh Torabi,^1^ Dan Tulpan,^2^ Istvan Rajcan,^1^ and Milad Eskandari^1*^**

^1.^ Department of Plant Agriculture, University of Guelph, Guelph, ON N1G 2W1, Canada

^2.^ Department of Animal Biosciences, University of Guelph, Guelph, ON N1G 2W1, Canada

**^*^**Corresponding author: Email: [meskanda@uoguelph.ca](file:///C:\Users\dtulpan\UofG%20Work\Papers\2019\Mohsen%20Y\meskanda@uoguelph.ca)

| **Table S1** Combined analyses of variances for yield in the 250 soybean genotypes across four tested environments (Ridgetown2018, Ridgetown2019, Palmyra2018, and Palmyra2019). | | | | | |
| --- | --- | --- | --- | --- | --- |
| **Fixed** | | | | | |
| **Effect** | **Num DF** | **Adj MS** | | **F-Value** | **P-Value** |
| Maturity | 1 | 95069527 | | 238.23 | 0.000 |
| Environment | 3 | 380200802 | | 777.64 | 0.000 |
| **Random** | | | | | |
| **Effect** | **Adj MS** | | **P-Value** | | |
| Replication | 146345973 | | 0.000 | | |
| Genotype | 1850778 | | 0.000 | | |
| Genotype × Environment | 490786 | | 0.001 | | |
| Heritability | 0.24 | |  | | |

| **Table S2** Combined analyses of variances for 390 nm in the 250 soybean genotypes across four tested environments (Ridgetown2018, Ridgetown2019, Palmyra2018, and Palmyra2019). | | | | | |
| --- | --- | --- | --- | --- | --- |
| **Fixed** | | | | | |
| **Effect** | **Num DF** | **Adj MS** | | **F-Value** | **P-Value** |
| Environment | 3 | 0.004674 | | 177.05 | 0.000 |
| **Random** | | | | | |
| **Effect** | **Adj MS** | | **P-Value** | | |
| Replication | 0.004309 | | 0.000 | | |
| Genotype | 0.000119 | | 0.000 | | |
| Genotype × Environment | 0.000095 | | 0.052 | | |
| Heritability | 0.57 | |  | | |
|  | | | | | |

| **Table S3** Combined analyses of variances for 550 nm in the 250 soybean genotypes across four tested environments (Ridgetown2018, Ridgetown2019, Palmyra2018, and Palmyra2019). | | | | | |
| --- | --- | --- | --- | --- | --- |
| **Fixed** | | | | | |
| **Effect** | **Num DF** | **Adj MS** | | **F-Value** | **P-Value** |
| Environment | 3 | 0.003267 | | 19.52 | 0.000 |
| **Random** | | | | | |
| **Effect** | **Adj MS** | | **P-Value** | | |
| Replication | 0.435220 | | 0.000 | | |
| Genotype | 0.003485 | | 0.000 | | |
| Genotype × Environment | 0.001167 | | 0.046 | | |
| Heritability | 0.46 | |  | | |
|  | | | | | |

| **Table S4** Combined analyses of variances for 660 nm in the 250 soybean genotypes across four tested environments (Ridgetown2018, Ridgetown2019, Palmyra2018, and Palmyra2019). | | | | | |
| --- | --- | --- | --- | --- | --- |
| **Fixed** | | | | | |
| **Effect** | **Num DF** | **Adj MS** | | **F-Value** | **P-Value** |
| Environment | 3 | 0.000015 | | 2.30 | 0.076 |
| **Random** | | | | | |
| **Effect** | **Adj MS** | | **P-Value** | | |
| Replication | 0.001293 | | 0.000 | | |
| Genotype | 0.000108 | | 0.000 | | |
| Genotype × Environment | 0.000007 | | 0.063 | | |
| Heritability | 0.85 | |  | | |
|  | | | | | |

| **Table S5** Combined analyses of variances for 730 nm in the 250 soybean genotypes across four tested environments (Ridgetown2018, Ridgetown2019, Palmyra2018, and Palmyra2019). | | | | | |
| --- | --- | --- | --- | --- | --- |
| **Fixed** | | | | | |
| **Effect** | **Num DF** | **Adj MS** | | **F-Value** | **P-Value** |
| Environment | 3 | 1.79665 | | 1056.42 | 0.000 |
| **Random** | | | | | |
| **Effect** | **Adj MS** | | **P-Value** | | |
| Replication | 1.05695 | | 0.000 | | |
| Genotype | 0.00207 | | 0.027 | | |
| Genotype × Environment | 0.00170 | | 0.998 | | |
| Heritability | 0.28 | |  | | |
|  | | | | | |

| **Table S6** Combined analyses of variances for 820 nm in the 250 soybean genotypes across four tested environments (Ridgetown2018, Ridgetown2019, Palmyra2018, and Palmyra2019). | | | | | |
| --- | --- | --- | --- | --- | --- |
| **Fixed** | | | | | |
| **Effect** | **Num DF** | **Adj MS** | | **F-Value** | **P-Value** |
| Environment | 3 | 0.00005 | | 2.02 | 0.425 |
| **Random** | | | | | |
| **Effect** | **Adj MS** | | **P-Value** | | |
| Replication | 0.115212 | | 0.000 | | |
| Genotype | 0.007613 | | 0.000 | | |
| Genotype × Environment | 0.000282 | | 0.085 | | |
| Heritability | 0.53 | |  | | |
|  | | | | | |
